# Time-series proteomic study of the response of HK-2 cells to hyperglycemic, hypoxic diabetic-like milieu

**DOI:** 10.1371/journal.pone.0235118

**Published:** 2020-06-24

**Authors:** Alberto Valdés, María Castro-Puyana, Coral García-Pastor, Francisco J. Lucio-Cazaña, María Luisa Marina

**Affiliations:** 1 Departamento de Química Analítica, Química Física e Ingeniería Química, Universidad de Alcalá, Alcalá de Henares, Madrid, España; 2 Instituto de Investigación Química Andrés M del Rio, IQAR, Universidad de Alcalá, Alcalá de Henares, Madrid, España; 3 Departamento de Biología de Sistemas, Universidad de Alcalá, Alcalá de Henares, Madrid, España; Anatomy, SWITZERLAND

## Abstract

During diabetes, renal proximal tubular cells (PTC) are exposed to a combination of high glucose and hypoxic conditions, which plays a relevant role in the development of diabetic kidney disease (DKD). In this work, a time-series proteomic study was performed to analyse the effect of a diabetic-like microenvironment induced changes on HK-2 cells, a human cell line derived from normal proximal tubular epithelial cells. Cells simultaneously exposed to high glucose (25 mM) and hypoxia (1% O_2_) were compared to cells in control conditions for up to 48 h. Diabetic conditions increased the percentage of death cells after 24 and 48 h, but no differences in the protein/cell ratio were found. The relative protein quantification using dimethyl-labeling and UHPLC-MS/MS analysis allowed the identification of 317, 296 and 259 proteins at 5, 24 and 48 h, respectively. The combination of statistical and time expression profile analyses indicated an increased expression of proteins involved in glycolysis, and a decrease of cytoskeletal-related proteins. The exposure of HK-2 cells to high glucose and hypoxia reproduces some of the effects of diabetes on PTC and, with the limitations inherent to *in vitro* studies, propose new mechanisms and targets to be considered in the management of DKD.

## Introduction

Proteins are key players in many biological processes and cellular signaling events, and the alteration of their abundancy can lead to the development of life threatening diseases [1]. It is also known that many proteins respond to different stimuli changing their expression in a time-dependent manner; therefore, protein expression monitoring over time is important to understand the mechanisms behind these diseases’ development. For protein identification and quantification, high-performance liquid chromatography coupled to mass spectrometry (HPLC-MS) has become a key technology due to its unbiased capacity where there is no need to decide which proteins to target in advance, and because thousands of proteins can be monitored at the same time and remarkable biological information can be accessed [2]. Diabetic kidney disease (DKD) is the most common cause of end-stage renal disease and it develops in approximately 40% of patients with diabetes [3]. Despite this fact, the pathological mechanisms leading to the development and progression of DKD remain poorly understood, and proteomics studies can help shedding some light. It has been demonstrated that increased blood concentrations of glucose and non-esterified fatty acids in diabetes are intracellularly reflected in the kidney, particularly in proximal tubules [4]. It is also evident that the proximal tubule is a prime mover in DKD so that it plays a critical role in the development and progression of DKD [5, 6]. In diabetes, high glucose (HG) content in proximal tubule cells (PTC) results from both increased glucose in plasma and also from excess glucose filtered by the glomeruli, resulting in an excessive glucose load in the tubule [5]. However, although a HG microenvironment is the primary causative factor for the development of DKD, hypoxia also plays a relevant role in the development and progression of this pathology [6–8]. This is particularly true for proximal tubules because they are especially vulnerable to hypoxia, and because PTC are propense to hypoxic injury in diabetes due to three relevant factors: i) diabetes increases two high energy consuming processes, namely gluconeogenesis and sodium reabsorption driven by Na^+^/K^+^-ATPase, in PTC (only their Na^+^/K^+^-ATPase consumes in basal conditions 80% of the O_2_ kidney supply) [9]; ii) diabetes leads to impaired O_2_ utilization because it induces mitochondrial dysfunction and alters substrate delivery in PTC; iii) diabetes reduces O_2_ delivery to PTC due to microvascular rarefaction [6].

Among others, the hypoxia inducible factor-1α (HIF-1α) protein orchestrates cellular adaptive responses to combat hypoxia, through the expression control of several proteins [10]. However, activation of the HIF system in the hypoxic diabetic kidney has been shown to be suboptimal [7, 11, 12] or even inexistent [8]. This may explain the renoprotective effect in DKD of HIF-1α activation by CoCl_2_ [8, 13] as well as the fact that a Pro582Ser polymorphism, which confers relative resistance of HIF-1α to the repressive effect of HG, is associated with DKD protection [14]. Activation of the HIF system under hypoxia has also been found to be consistently supressed by HG in PTC [15], and this deficient response seems to be specific for PTC: HIF-1α expression in primary human mesangial cells increased under hypoxia, but a further increase in its expression was observed when these cells were simultaneously exposed to HG and hypoxia [16]. On the other hand, human PTC and HeLa cells did not respond to HG, and in mouse MC, HIF-1α was induced by HG under normoxic conditions, whereas in mouse PTC, that response was absent. In the same way, glucose promotes in cancer cells the expression of HIF-1α [17]. We have previously set up an *in vitro* model of diabetes-induced proximal tubular dysfunction in which HK-2 cells, a human cell line derived from normal proximal tubular epithelial cells, are exposed to a diabetic-like microenvironment consisting of HG (25 mM) and hypoxia (1% O_2_). In these conditions, HIF-1α fails to stabilize and accumulate in HK-2 cells (i.e. the same defective response found in the hypoxic diabetic kidney) [18]. Furthermore, impaired HIF-1α regulation in cells exposed to hyperglycemic, hypoxic diabetic-like milieu led to diminished production of vascular endothelial growth factor-A and inhibition of cell migration (responses respectively involved in tubular protection and repair). These effects, as well as impaired HIF-1α regulation, were reproduced in normoglycemia in HK-2 cells incubated with microparticles released by HK-2 cells exposed to hyperglycemic, hypoxic diabetic-like milieu [18]. Finally, neither HG alone nor hypoxia alone affected the migration of HK-2 cells [18] and the release of microparticles under hyperglycemia was greatly diminished in the absence of hypoxia (unpublished results). In summary, HK-2 cells exposed simultaneously to hyperglycemia and hypoxia (i.e. two major conditions of the microenvironment that surrounds proximal tubular cells in diabetes)-but not HK-2 cells exposed separately to either condition- show functional alterations that reproduce some specific effects of diabetes on proximal tubular cells. Given that the monitorization of untargeted protein evolution in HK-2 under hyperglycemic/hypoxic conditions is currently unknown, in the present work we have conducted a shotgun time-series proteomic study based on UHPLC-MS/MS together with stable isotope dimethyl labeling (DML) to relatively quantify the changes in protein expressions over time. The results obtained propose new mechanisms and targets to be investigated in the future for a better understanding of DKD.

## Materials and methods

### Chemicals and reagents

All reagents herein used were of analytical grade or higher. Acetic acid (AA), ACN, acetone, ethyl-acetate, methanol and urea were obtained from Scharlau (Barcelona, Spain). Formic acid (FA) was from Fisher Scientific (Geel, Belgium). Ammonia solution and NaCl were obtained from Merck (Darmstadt, Germany). β-glycerophosphate, DTT, EDTA, formaldehyde (CH_2_O) (37%, v/v), iodoacetamide (IAA), n-octyl-β-d-glucopyranoside (BOG), PBS, protease inhibitor cocktail, sodium cyanoborohydride (NaBH_3_CN), sodium fluoride (NaF), sodium metavanadate (NaVO_4_), tributylphosphate, triethylammonium bicarbonate (TEAB) and TFA were purchased from Sigma-Aldrich (St. Louis, MO). Trypsin/Lys-C mix (Mass Spec grade V5072) was purchased from Promega (Madison, WI), deuterated formaldehyde (CD_2_O) (20%, v/v) was obtained from ISOTEC (Miamisburg, OH) and sodium pyrophosphate from Alfa Aesar (Kandel, Germany). Ultrapure water was prepared by Milli-Q water purification system (Millipore, Bedford, MA).

### Cell culture conditions

Human proximal tubular epithelial (HK-2) cells were purchased from the American Type Culture Collection (Rockville, MD, USA). HK-2 cells were maintained in DMEM/F12 (ThermoFisher, Grand Island, NY, USA) supplemented with 10% FBS, 1% penicillin/streptomycin/amphoterycin B, 1% glutamine and 1% insulin-transferrine-selenium (ITS) (ThermoFisher, Grand Island, NY, USA). One week before the beginning of the experiments, cell culture medium was changed to DMEM normal glucose (NG, 5.5 mM glucose, ThermoFisher, Grand Island, NY, USA) supplemented as stated above. Cells were routinely maintained in 21% O_2_ (normoxic conditions) at 37 °C. To perform the experiments, seven p35 cultured plates were seeded with 2.5 x 10^5^ cells (90% confluence) per time and treatment condition. When completely attached, cells were exposed to either medium DMEM NG or DMEM high glucose (HG, 25 mM glucose, ThermoFisher, Grand Island, NY, USA) supplemented with 0.5% FBS, 1% penicillin/streptomycin/amphoterycin B, 1% glutamine and 1% ITS under hypoxic (1% O_2_) or normoxic conditions (21% O_2_), for 5, 24 and 48 h. For 48 h experiments, cell culture medium was refreshed at 24 h. Hypoxia experiments were performed in an In vivo200 hypoxia workstation (Ruskinn Technology, West Yorkshire, UK). Cells were manipulated and lysed inside the chamber, and all media and buffers were pre-equilibrated to 1% O_2_. Three replicates were used to determine the total cell number and the cell viability based on the Trypan Blue dye exclusion assay, and a hemocytometer. Briefly, cells were detached by trypsinization and, together with the detached cells recovered from the culture medium, they were stained with Trypan Blue (1:1). Then, white (viable) and blue (dead) cells were counted using a light microscope and a hemocytometer. For the proteomic study, and after incubation, the growth medium from three culture plates was removed by aspiration, washed with PBS, and proteins were directly extracted from attached cells. An additional plate per time and treatment condition was used to check by Western blot analysis the interaction between HG and hypoxia resulting in specific loss of hypoxia-induced accumulation of HIF-1α in HK-2 cells [18]. Statistical analysis was performed using Statistica software version 7.1 (StatSoft, Inc., USA). One-way ANOVA with LSD Post-hoc test was employed to determine any significant differences between mean values using p < 0.05.

### Sample preparation for UHPLC–MS/MS-based proteomics

Cells were lysed with 300 μL of lysis buffer (6 M urea, 1% (m/v) BOG, 0.15 M NaCl, 1.3 mM EDTA, 1 mM NaVO_4_, 5 mM NaF, 2.5 mM sodium-pyrophospate, and 5 mM β-glycerophosphate in PBS) supplemented with 10 μL of protease inhibitor cocktail to prevent protein degradation. The samples were incubated for 60 min at 4 °C, sonicated for 30 min at 0 °C in a water bath, and then centrifuged at 10000g at 4 °C for 15 min. The supernatants were collected, and protein concentration was quantified using Bradford assay (Bio-Rad Laboratories, Hercules, CA). 20 μg of proteins were mixed with 500 μL of an ice-cold tributylphosphate/acetone/methanol mixture (1:12:1, v/v/v) and incubated at 4 °C for 90 min (mixing every 15 min). The precipitate was centrifuged for 15 min (3000g at 4 °C), washed with 1 mL of cold acetone, and air-dried. Pellets were dissolved in 20 μL of 1% (w/v) BOG with 20% (v/v) ACN in 0.1 M TEAB, and the proteins were reduced with 10 μL of 45 mM DTT at 56 °C for 15 min, cooled down to room temperature and alkylated with 10 μL of 100 mM IAA for 15 min in the dark. Samples were then digested with 0.5 μg of LysC/trypsin solution (5% (w/w), of total protein content) and incubated at 37 °C overnight in darkness. The digests were dried to remove ACN and resuspended in 70 μL of 0.1 M TEAB, and BOG was extracted with water-saturated ethyl-acetate [19]. Dimethyl labelling was performed according to Boersema et al [20]. Briefly, tryptic peptide mixtures were reconstituted in TEAB and NG-Normoxia samples were labelled as light (L) using regular formaldehyde (CH_2_O, 4% (v/v)), and HG-Hypoxia samples were labelled as medium (M) using deuterated formaldehyde (CD_2_O, 4% (v/v)). Technical replicates were included for each sample in a swap-labelling experiment form (NG-Normoxia samples labelled as M, and HG-Hypoxia samples labelled as L). To control the labelling efficiency and optimize the UHPLC peptide loading quantity and MS parameters, 120 μg of NG-Normoxia samples were divided into six aliquots of 20 μg, and three aliquots were labelled as L, and the other three as M, following the same procedure as explain above. These samples were considered as “Test” samples. After a brief vortexing, 4 μL of freshly prepared 0.6 M NaBH_3_CN solution was added to each sample, and the vials were incubated for 60 min at room temperature with mild agitation. The reaction was terminated by adding 1% (v/v) ammonia solution (Merck, Germany), and 5% (v/v) FA (Merck, Germany) to consume the excess labelling reagents. Finally, the real samples labelled as L and M were mixed together and for the “Test” samples, one L sample and one M sample were mixed together, generating 3 “Test” samples. All mixed samples were desalted on Isolute C18 solid-phase extraction columns as specify by the manufacture (1 mL, 50 mg capacity, Biotage, Uppsala, Sweden). After being desalted, peptides were dried in a SpeedVac and redissolved in 2% (v/v) ACN and 0.1% (v/v) TFA prior to UHPLC–MS/MS analysis. Two replicates were analysed for each “Test” condition, and six replicates (considering biological and technical replicates) were analysed for the real samples at each time point, giving a total of 18 LC-MS/MS runs.

### UHPLC–MS/MS analysis

Aliquots of 5 μL containing ∼ 1, 2, 4 or 6 μg (for “Test” samples), or ∼ 6 μg (for real samples) tryptic peptides were injected into a LC–MS/MS system consisting of an UltiMate 3000 quaternary UHPLC (Thermo Fisher Scientific, Bremen, Germany) coupled to a Q Exactive Pro Mass Spectrometer (Thermo Fisher Scientific, Bremen, Germany) using a HESI-II probe at 3 kV. Peptide separations were performed on a C18 Ascentis Express column (Sigma, St Louis, USA), having 100 × 2.1 mm i.d. dimensions (fused-core^®^ particles with 0.5 μm thick porous shell and an overall particle size of 2.7 μm), and a guard column (5 × 2.1 mm i.d. × 2.7 μm particle size) of the same composition as the analytical columns. Columns were kept at 55◦C during the analysis of the samples. The separations were performed at a flow rate of 0.3 mL/min with mobile phases A (2% (v/v) ACN, 0.3% (v/v) AA, 0.1% (v/v) FA) and B (80% (v/v) ACN, 0.3% (v/v) AA, 0.1% (v/v) FA). A 60 min gradient from 0.2% B to 46.2% B was used. The mass spectrometer was operated in positive-ion mode and a Top6 data-dependent acquisition (DDA) mode was used. MS1 spectra were acquired within m/z 350–1600 with an automatic gain control (AGC) of 3 x 10^6^ at 35,000 resolution and with a maximum ion time (IT) of 110 ms. Up to 6 of the most abundant precursor ions with ≥2 and ≤8 charges and threshold intensity of 2 x 10^4^ were selected for higher-energy C-trap dissociation (HCD). An isolation window of 2 m/z and normalized collision energy of 28 were used. Ions that were once selected for acquisition were dynamically excluded for 20 s for further fragmentation. MS2 spectra were acquired with an AGC of 1 x 10^5^ at resolution of 35,000 and a maximum IT of 110 ms. A Top12 DDA mode was also tested, keeping the MS1 parameters as for the Top6 but increasing the number of precursor ions to 12, and acquiring the MS2 spectra with an AGC of 1 x 10^5^ at resolution of 17,500 and a maximum IT of 50 ms.

### MaxQuant analysis

All MS raw files were collectively processed with MaxQuant (version 1.6.3.3) [21] using the Andromeda search engine [22]. The FDR was set to 1% for both proteins and peptides, and a minimum of two peptides with at least seven amino acid length was specified. MaxQuant scored peptides for identification based on a search with a maximal precursor mass deviation of 20 ppm (first search) and 4.5 ppm (main search), and fragment masses deviations of up to 20 ppm. The Andromeda search engine was used for the MS/MS spectra search against a concatenated forward and reversed version of the Uniprot human database (downloaded on April 24^th^, 2019 and containing 20,428 entries). Trypsin was selected as digestive enzyme and a maximum of two missed cleavages were allowed. Carbamidomethylation of cysteine residues was set as the fixed modification, and the oxidation of methionine and protein N-acetylation were set as variable modifications. For dimethyl labeling, DimethylLys0 and DimethylNter0 were set as light labels, and DimethylLys4 and DimethylNter4 were set as medium labels. A minimum peptide ratio count of two and at least one “razor peptide” was required for quantification. All mass spectrometry proteomics data and search results have been deposited to the ProteomeXchange Consortium via the PRIDE [23] partner repository with the data set identifier PXD015102.

### Database search/data processing

Prior to any statistical analysis, identifications flagged as reverse, potential contaminants, or proteins identified only by site modification were excluded for further analysis, and the normalized relative protein abundance was transformed to the log2 scale. For PCA, only proteins present in all biological and technical replicates across the three time points were considered to avoid imputation of missing values. Thereafter, the values were normalized (subtracting the mean value of each data set and dividing by its standard deviation), and the temporal profiles were clustered using the fuzzy c-means clustering algorithm included in the Mfuzz toolbox [24]. Different combinations of cluster sizes and fuzzification parameters were explored and found optimal partitioning with c = 6 and m = 3. The lists of proteins with Euclidean distance larger than 0.5 in each cluster were searched for enriched gene ontology (GO) terms or pathways using the web-based PANTHER (http://www.pantherdb.org/) [25] or STRING [26]. The corrected p-values were derived from a Fisher's Exact test followed by Benjamini and Hochberg false discovery rate correction, and considered significant when < 0.05. Thereafter, the most significantly altered proteins between hypoxia/hyperglycemia against normoxia/normoglycemia were identified. For this analysis, a protein was included in the analysis when it was quantified in at least four sample replicates at the different time points. In these cases, a p value of < 0.05 (after one sample t-test) and a 1.2-fold change cut-off filter (equivalent to 0.26 and -0.26 in log2 scale) were applied. Finally, the list of significantly altered proteins at 48 h was searched for enriched GO biological processes using PANTHER and STRING softwares using the same parameters as previously specified.

## Results

### Cell viability and protein content

The main objective of this work was to carry out a time-course proteomic study to investigate the changes induced by a diabetic-like hyperglycemic/hypoxic medium in HK-2 cells, a cell line derived from human proximal tubular cells, as an *in vitro* model of the effects of the diabetic milieu on proximal tubular cells in the context of DKD. For this study, equal number of cells (5 × 10^5^ per mL) were plated at 90% confluence in p35 cultured dishes and when completely attached, they were treated for 5, 24 and 48 h with HG (25 mM) medium in hypoxic conditions (1% O_2_), or with NG medium (5.5 mM) in normoxic conditions (21% O_2_). The interaction between HG and hypoxia resulting in specific loss of hypoxia-induced accumulation of HIF-1α in HK-2 cells, which has important pathological implications in the context of proximal tubular disfunction in DKD [18], was routinely checked (results are not shown).

**Fig 1:**
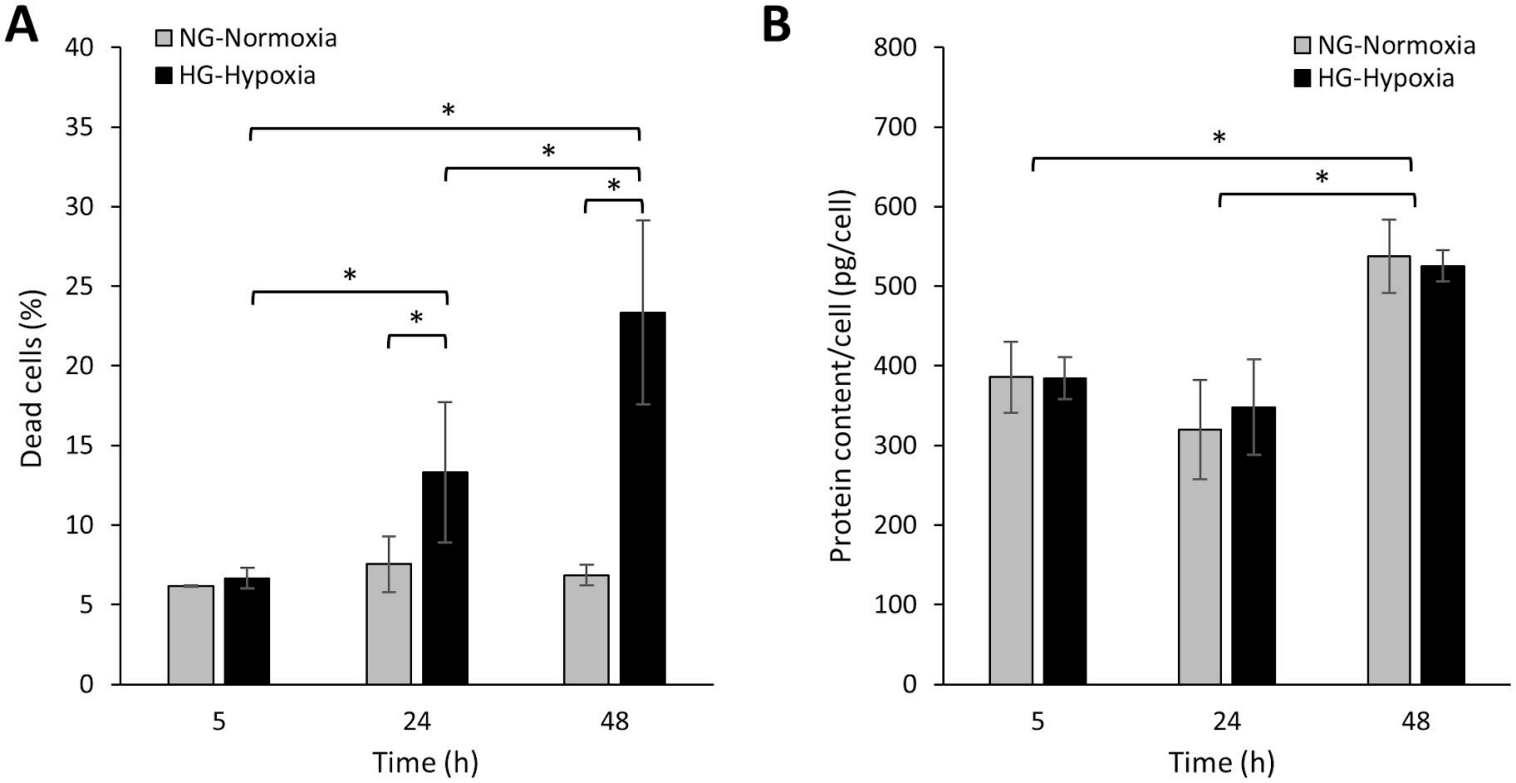
Percentage of dead cells (A) and protein content/cell (B) after incubation of HK-2 cells in NG (5.5 mM glucose)-normoxia (21% O_2_) or HG (25 mM glucose)-hypoxia (1% O_2_). Error bars are given as the standard deviation of three independent experiments. *Asterisks indicate significant differences between samples after ANOVA with LSD Post-hoc, p-value < 0.05.

While the percentage of death cells increased from 7% to 12% after 24 h in HG-Hypoxia compared to NG-Normoxia, and rose up to more than 20% after 48 h (Fig 1A), no differences in the protein/cell ratio between the two experimental groups were found at any time point (Fig 1B).

### Peptide loading quantity and MS acquisition

The proteomic study was performed using preliminary UHPLC and MS conditions as previously reported by Lenčo et al. [27] and some parameters were then optimized. Firstly, the UHPLC peptide loading quantity was optimized based on the total number of identified/quantified proteins after loading 1, 2, 4 and 6 μg of the peptides from the “Test” samples, and the labelling efficiency was checked (Table 1). As shown in this table, the number of identified/quantified proteins increased when more peptides were injected, and the use of the Top6 MS acquisition method allowed the quantification of more than 270 proteins after the injection of 6 μg. Secondly, a Top12 MS acquisition method was evaluated injecting 6 μg of proteins, which did not improve the results obtained using the Top6 method. Moreover, the variability for the protein quantification was similar for all the preparations, indicating that the peptide labelling was adequate. Compared to the work carried out by Lenčo et al. [27] a lower number of quantified proteins was expected as those authors used a faster mass spectrometer and a smaller inner diameter analytical column, which decreases the sample dilution and increases the number of identified proteins. However, they did not quantify and only identified proteins, without specifying the number of peptides used for this identification. In the present study, a restrictive criterion was applied and proteins were identified when at least two peptides belonging to each protein were detected. In addition, a minimum peptide ratio count of two was selected for protein quantification, which means that two peptides for each labelled sample were needed. Despite the fact that the total number of quantified proteins could be increased if more peptides/proteins are injected, the protein quantity was set to 6 μg to analyse the real samples to avoid column overloading and to prevent MS instrument contamination.

**Table 1:** Loaded peptide quantity, MS detection method, identified/quantified proteins and average quantified proteins in HK-2 cells after UHPLC-Orbitrap-MS/MS analysis.

Loaded Peptide Quantity (μg)	MS Method	Identified proteins	Quantified proteins	Protein quantification Average ± SD Ratio
1	Top6	51 ± 2	32 ± 1	0.93 ± 0.13
2	Top6	131 ± 5	81 ± 4	0.97 ± 0.18
4	Top6	265 ± 4	188 ± 4	0.96 ± 0.14
6	Top6	321 ± 1	278 ± 4	0.96 ± 0.15
6	Top12	207 ± 1	137 ± 4	0.96 ± 0.16

### Overview of the temporal expression protein changes

**Fig 2:**
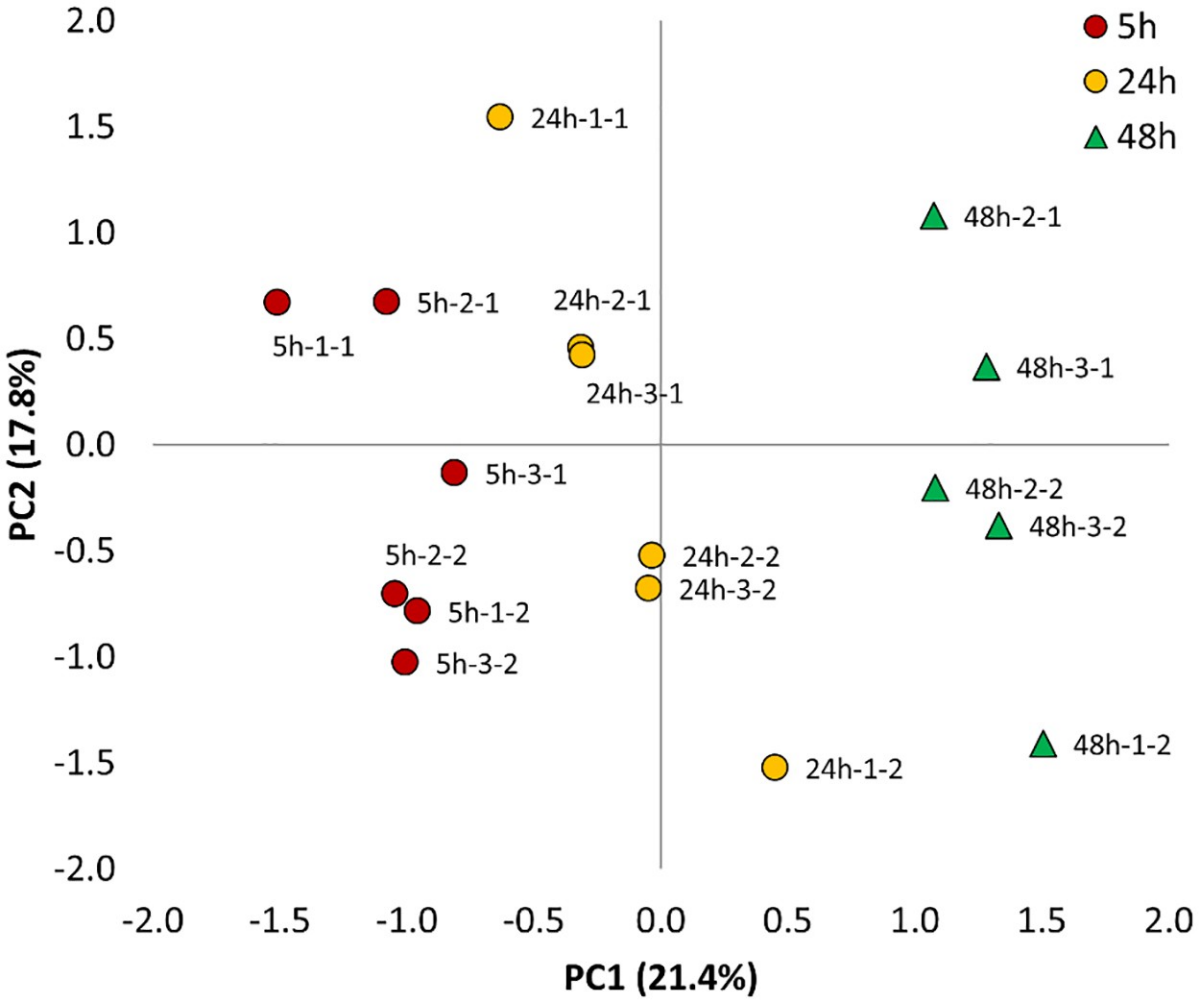
Principal component analysis of the 150 quantified proteins in all biological and technical replicates across the three time points (5, 24 and 48 h) in HK-2 cells treated with HG (25 mM glucose)-hypoxia (1% O_2_) in comparison to NG (5.5 mM glucose)-normoxia (21% O_2_). The first number after the different time points indicates the biological replicate, and the second number indicates the technical replicate.

Based on the above information, 6 μg of peptides were injected and the Top6 MS method was selected to study the effect of HG-Hypoxia against NG-Normoxia over time. After applying a FDR of 1%, a total number of 526 proteins were identified, with an average of 317 ± 64, 296 ± 25 and 259 ± 54 proteins at 5, 24 and 48 h, respectively (Table 2 and S1 Table). In addition, a total of 150 proteins were simultaneously quantified in all biological and technical replicates, and a PCA multivariate analysis was carried out to determine, compare and visualize the overall relation between the three conditions studied (Fig 2). This analysis indicates that the combination of the two main components captured almost 40% of the variance of the data, being the principal component 1 the one that clearly separates and groups together the samples representing 5, 24 and 48 h.

**Table 2:** Number of quantified proteins after the incubation of HK-2 cells in NG-Normoxia and HG-Hypoxia conditions.

Parameter	5 h	24 h	48 h
Proteins in NG-Normoxia (L)/HG-Hypoxia (H)	315	277	250
Proteins in HG-Hypoxia (L)/NG-Normoxia (H)	319	314	269
Average proteins (considering 6 replicates)	317	296	259
Proteins simultaneously quantified in all replicates	150		
Proteins simultaneously quantified in at least four replicates	286	282	243
Proteins in at least four replicates with p-val ≤ 0.05	35	60	59
Up-regulated proteins^a^	2	3	14
Down-regulated proteins^b^	2	8	13

^a^ p-val ≤ 0.05 and log2 fold change ≥ 0.26 between HG-Hypoxia vs NG-Normoxia.

^b^ p-val ≤ 0.05 and log2 fold change ≤ -0.26 between HG-Hypoxia vs NG-Normoxia.

**Fig 3:**
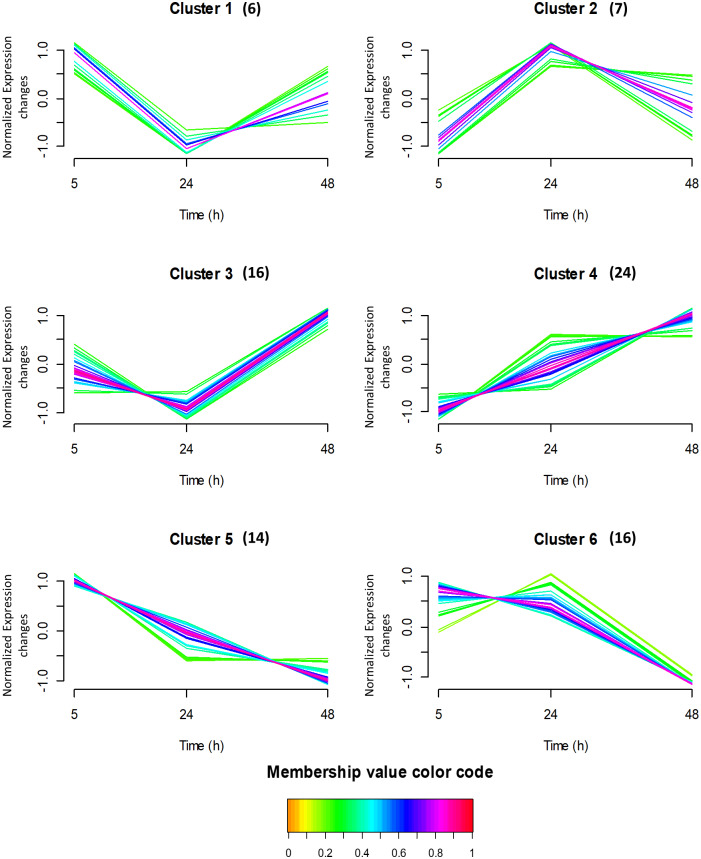
Time-course protein profiles obtained by fuzzy c-means clustering in HK-2 cells treated with HG (25 mM glucose)-hypoxia (1% O_2_) in comparison to NG (5.5 mM glucose)-normoxia (21% O_2_) at 5, 24 and 48 h. The y axis is log10 transformed and normalized, and the number of proteins considered in each cluster is given in parentheses. Each trace is colour coded according to its membership value for the respective cluster (see colour bar).

Thereafter, the expression of the same 150 proteins used for PCA were searched for patterns in their time expression profiles using the fuzzy c-means clustering algorithm. This algorithm does not establish a protein expression cut-off filter, but it measures the expression similarity (based on the distance, connectivity, and intensity) between the different proteins across the time, and then groups those proteins accordingly. It has to be noted that each protein can belong to more than one cluster. Using this software, the proteins were clustered in six profiles, containing between 6 and 24 proteins (Fig 3). In the figure, each trace corresponds to a protein, and the colour is coded according to its membership value for the respective cluster (higher values indicate higher similarity to the stablished profile). The proteins in each cluster were then analysed (by functional enrichment analyses) to identify overrepresented GO term and pathways using PANTHER (S2 Table) and STRING (S3 Table) softwares. The proteins considered in cluster 1 (with decreased expression between 5 h and 24 h, but slightly increased between 24 and 48 h) are involved in the translational initiation biological process as some proteins are structural constituent of ribosome. In cluster 2, the protein expression dramatically increased between 5 h and 24 h and then it slightly decreased between 24 and 48 h. These proteins are involved in several terms, but the main molecular function is the RNA binding. The expression of proteins considered in cluster 3 decreased between 5 h and 24 h, but it dramatically increased between 24 and 48 h. These proteins are involved in the broad molecular functions RNA binding and processing. Cluster 4 was the one with the highest number of proteins (24), which abundance continuously increased from 5 to 48 h. These proteins are involved in several biological processes (glycolytic process, ATP generation from ADP, pyruvate biosynthetic process, NADH regeneration, canonical glycolysis), in the Glycolysis/Gluconeogenesis pathways (ENO1, PKM, PGAM1, ALDOA, TPI1, PGK1 and GAPDH) or in the HIF-1 signaling pathway. On the other hand, proteins represented in cluster 5 were highly abundant at 5 h, and their expression constantly decreased with time, being minimal at 48 h. These proteins are involved in the actin-dependent ATPase activity, they are structural constituents of cytoskeleton, or they belong to the ubiquitin-like protein ligase binding, the cadherin binding, or the identical protein binding molecular functions. Some of them also form part of different pathways such as the Cytoskeletal regulation by Rho GTPase. Finally, the expression of proteins in cluster 6 was constant between 5 and 24 h, but it decreased after 48 h. These proteins form part of the structural molecule activity and the RNA binding functions, and in the Eukaryotic Translation Elongation pathway. In summary, this analysis confers an overview of the protein expression profiles and the functions in which the proteins are involved, without losing the information of proteins with small but similar expression patterns.

### Significantly altered proteins at specific time points

**Fig 4:**
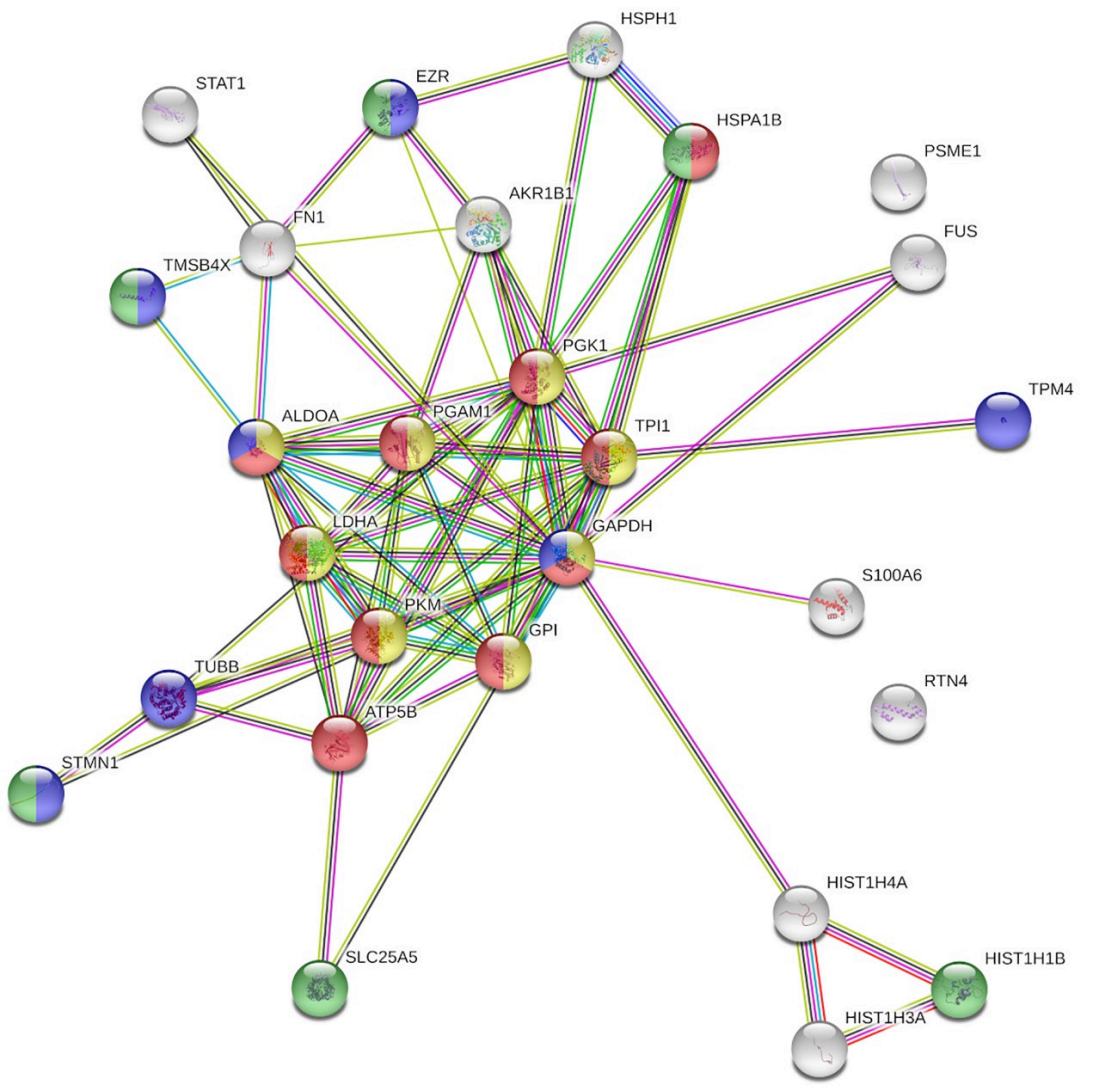
STRING diagram representing protein interaction pathway derived from the 27 proteins significantly altered at 48 h after the incubation of HK-2 cells with HG-Hypoxia in comparison to NG-Normoxia. The average local clustering coefficient as reported by STRING is 0.703. Proteins in yellow belong to the glycolytic process; proteins in red belong to the ATP metabolic process; proteins in blue belong to the cytoskeleton organization; and proteins in green belong to the regulation of organelle organization.

In addition to the multivariate and the temporal expression analyses, the most significantly altered proteins between HG-Hypoxia and NG-Normoxia at each specific time point were identified. For this analysis, several filtering criteria were applied (Table 2). Firstly, a protein was selected for further processing when it was identified in at least four sample replicates at the different time points. Taking into account this filtering criteria, 286, 282 and 243 proteins were quantified at 5, 24 and 48 h respectively. Secondly, a p-value cut-off filter <0.05 after one sample t test analysis was applied, and among the proteins quantified at each time point, 35, 60 and 59 passed this criteria at 5, 24 and 48 h, respectively. Finally, and to identify the most significantly altered proteins, a 1.2-fold change cut-off filter (equivalent to 0.26 and -0.26 in log2 scale) was applied and only 4, 11 and 27 proteins (at 5, 24 and 48 h, respectively) passed this criteria. The full list of proteins and their expression values are shown in (Table 3). Additionally, the list of significantly altered proteins at 48 h was searched for enriched GO biological processes using PANTHER and STRING softwares, and the results are shown in Table 4 and Fig 4. The results show that the expression of two ribosomal proteins was decreased (RPL18, RPL34) while the expression of two histone subunits was increased (HIST1H3A, HIST1H4A) after 5 h of treatment. After 24 h, the expression of three additional ribosomal-related proteins was decreased (RPS27, RPL28, UBA52), as well as the expression of different cytoskeletal-related proteins (CNN2, STMN1, TPM1), or other proteins (FN1, STAT1). On the other hand, the levels of two proteins involved in the glycolysis pathway were increased (GPI, LDHA), as well as the rate-limiting enzyme implicated in the reduction of glucose to sorbitol via the polyol pathway (AKR1B1). At 48 h, the levels of two histone subunits already were observed as increased at 5 h (HIST1H3A and HIST1H4A), and the levels of the subunit HIST1H1B, were observed as decreased, while the expression of some proteins observed as decreased at 24 h, decreased even more (FN1, STAT, STMN1). Moreover, the expression of three additional cytoskeletal related proteins were observed as decreased (TMSB4X, TPM4, TUBB), as well as the proteasome subunit PSME1, the ADP/ATP Translocase 2 (SLC25A5) and the ATP Synthase F1 Subunit Beta (ATP5B). On the other hand, most of those proteins with positive ratios are involved in the glycolysis pathway (ALDOA, GAPDH, GPI, LDHA, PGAM1, PGK1, PKM, TPI1), or they are associated with the endoplasmic reticulum (RTN4, HSPA1B, HSPH1). Finally, the expression of AKR1B1 was observed as increased, being its expression higher than at 24 h.

**Table 3:** Significantly altered proteins after the incubation of HK-2 cells with HG-Hypoxia in comparison to NG-Normoxia for 5, 24 and 48 h.

	5 h			24 h			48 h		
Gene name	log2FC	N° samples	p-value	log2FC	N° samples	p-value	log2FC	N° samples	p-value
AKR1B1	-0.13	6	1.9E-03	0.27	6	1.2E-02	0.52	6	2.0E-03
ALDOA	-0.01	6	5.7E-01	0.25	6	1.2E-05	0.45	6	4.9E-04
ATP5B	-0.10	6	2.7E-01	-0.12	6	9.4E-02	-0.29	6	2.2E-02
CNN2	-0.11	6	1.8E-01	-0.27	6	1.2E-03	-0.46	2	3.6E-01
EZR	0.03	6	5.7E-01	0.08	6	1.3E-01	0.36	6	6.8E-04
FN1	-0.03	6	2.6E-01	-0.65	6	5.9E-04	-0.92	6	6.7E-07
FUS	-0.02	6	6.4E-01	-0.02	6	4.6E-01	0.30	5	1.3E-03
GAPDH	-0.01	6	7.4E-01	0.07	6	1.1E-01	0.27	6	1.1E-02
GPI	-0.09	6	7.5E-02	0.28	6	5.0E-03	0.46	5	2.7E-03
HIST1H1B	0.03	6	5.7E-01	-0.09	6	2.3E-01	-0.49	5	1.1E-02
HIST1H3A	0.58	6	4.7E-02	-0.11	6	2.5E-01	-0.66	6	1.1E-03
HIST1H4A	0.60	6	4.0E-02	-0.11	6	1.7E-01	-0.57	6	9.7E-03
HSPA1B	0.22	6	4.1E-04	0.23	6	1.6E-04	0.37	6	4.0E-04
HSPH1	-0.08	2	2.7E-01	0.53	2	1.3E-01	0.75	4	2.7E-04
LDHA	-0.01	6	9.1E-01	0.43	6	1.5E-05	0.85	6	1.5E-05
PGAM1	-0.01	6	7.0E-01	0.18	6	4.0E-03	0.40	6	5.0E-04
PGK1	-0.04	6	2.0E-01	0.23	6	7.0E-05	0.60	6	4.0E-05
PKM	-0.03	6	1.3E-01	0.12	6	2.2E-03	0.28	6	2.9E-03
PSME1	0.03	6	7.2E-01	-0.10	6	5.3E-02	-0.29	4	1.5E-02
RPL18	-0.30	6	6.3E-03	-0.22	6	3.9E-02	0.02	5	7.4E-01
RPL28	-0.30	2	1.4E-01	-0.40	6	7.6E-03	-0.37	5	1.2E-01
RPL34	-0.34	6	2.0E-04	-0.14	6	3.3E-02	-0.01	6	9.0E-01
RPS27	0.10	6	4.4E-01	-0.36	4	3.4E-02	-0.04	3	8.8E-01
RTN4	-0.01	6	8.6E-01	0.14	6	1.2E-01	0.37	5	1.7E-02
S100A6	0.01	6	9.1E-01	-0.18	6	7.6E-02	-0.30	6	1.5E-05
SLC25A5	0.15	6	1.4E-02	-0.03	6	5.5E-01	-0.33	6	8.9E-03
STAT1	0.05	6	1.8E-02	-0.29	6	9.0E-04	-0.79	6	1.4E-06
STMN1	-0.14	6	1.7E-01	-0.26	6	4.7E-04	-0.70	6	4.3E-03
TMSB4X	0.09	6	5.8E-01	-0.18	6	2.2E-02	-0.40	6	1.7E-03
TPI1	-0.05	6	8.7E-02	0.21	6	9.6E-06	0.35	6	4.0E-04
TPM1	-0.13	6	1.2E-01	-0.90	4	7.8E-06	-	-	-
TPM4	-0.05	6	4.8E-01	-0.10	6	1.5E-01	-0.31	6	3.9E-02
TUBB	-0.15	6	5.9E-03	-0.24	6	5.2E-03	-0.29	6	1.0E-03
UBA52	-0.14	6	5.2E-02	-0.33	6	6.7E-04	-0.13	6	3.8E-02

* In grey, significantly altered proteins.

**Table 4:** Overrepresented PANTHER GO biological processes after the incubation of HK-2 cells with HG-Hypoxia in comparison to NG-Normoxia for 48 h.

PANTHER GO Biological Process	N° proteins (reference)	48 h			
		N° proteins	Protein names	Enrichment	FDR
Glycolytic process	18	5	PGK1, ALDOA, PKM, GPI, TPI1	> 100	1.4e-07
Glucose metabolic process	25	3	PGK1, GPI, TPI1	96.24	7.1e-04
Regulation of proteasomal protein catabolic process	25	2	HSPA1B, PSME1	64.16	3.8e-02
Carbohydrate biosynthetic process	43	3	PGK1, GPI, TPI1	55.95	2.7e-03
Oxidation-reduction process	185	5	PGK1, ALDOA, PKM, GPI, TPI1	21.67	4.9e-04
Regulation of organelle organization	271	4	HIST1H1B, TMSB4X, HSPA1B, STMN1	11.84	2.7e-02
Cytoskeleton organization	587	5	TMSB4X, TPM4, HSPA1B, STMN1, TUBB	6.83	4.7e-02

## Discussion

In this study, a UHPLC-MS method was optimized to monitor, for the first time, the protein expression dynamics of HK-2 cells subjected to a diabetic-like microenvironment consisting of HG (25 mM) and hypoxia (1% O_2_) at different time points (5, 24 and 48 h). One important aspect when studying proteome dynamics is that changes in protein abundance depend on the synthesis of new proteins and/or their degradation, which often requires hours/days. However, the analysis time-frame of these *in vitro* proteomics studies is usually limited because the control of cell characteristics is commonly lost after 72 hours [28]. The present study demonstrates that short exposure times (5 h) do not significantly affect the overall protein expression, but this expression is significantly affected when the exposure time is sustained for 24 and 48 h, being the changes at 48 h the most remarkable.

As presented in the results section, the most significant protein changes are related to the glycolytic pathway. It is known that oxidation of glucose in the cytosol through glycolysis leads to pyruvate as a final product and renders only two ATP molecules (as compared to 38 ATP molecules generated by complete oxidation of glucose via pyruvate through the tricarboxylic acid cycle and oxidative phosphorylation). Nevertheless, the rate of glucose metabolism through this pathway, in which oxygen is not required, is 10–100 times faster than that through the complete aerobic oxidation of glucose in mitochondria [3]. Therefore, anaerobic glycolysis in the hypoxic kidneys of diabetic patients might contribute to the quick metabolism of glucose from the systemic circulation [29]. In fact, previous studies have shown that the development of DKD is associated with increased anaerobic glycolysis in detriment of complete aerobic oxidation of glucose in mitochondria [30, 31]. In this context, it is not surprising that, in the specific conditions performed in the present study, the glycolysis pathway is the most affected pathway over time in HK-2 cells, being increased from 5 to 48 h. The enhanced expression of 7 glycolytic enzymes (ALDOA, ALDOB, ENO1, GAPDH, PGAM1, PKM2 and TPI1), as well as LDHB and AKR1B1 was identified in a proteomic analysis of post-mortem glomeruli of kidneys from diabetic patients, [31], which is highly coincident with the data obtained in our study. This suggests that our experimental *in vitro* model of diabetic-like milieu-induced protein expression changes in PTC reproduces important changes in the expression of glucose metabolizing enzymes induced by diabetes at the renal level, and that some of the observed changes in our setting might be beneficial adaptive responses to the diabetic-like microenvironment.

A previous study in db/db type 2 diabetic mice shows little coincidence with our proteomic results. In that study, transcriptomic and metabolomics analyses also indicated that diabetes increases the expression of several glycolytic enzymes in kidney cortex, which is 90% proximal tubules [30], but the enzymes whose mRNA expression was significantly increased were hexokinase, phosphofructokinase and pyruvate kinase (the only enzyme coincident with our study). The little correlation between the proteomic and the transcriptomic profiles has been previously found in cultured skin fibroblasts from type 1 diabetes patients, and suggested to the authors that the expression of glycolytic enzymes is mainly regulated at a post-transcriptional level [32]. A similar discordance between the transcriptome and the metabolome in sciatic nerve from db/db type 2 diabetic mice led the authors to suggest that post-transcriptional or post-translational modifications are important in the development of diabetic nephropathy [30]. Moreover, different transcriptomic studies of kidney biopsy samples from patients with DKD or DN have not identified significant changes in the mRNA levels of glycolytic enzymes [33, 34].

Besides glycolytic enzymes, other proteins whose changes might be considered as part of a beneficial adaptive response to the diabetic-like microenvironment are STAT1 and HSPs. Inhibition of STAT1 (as in our results), a cytosolic transcription factor, has been found to protect against tubulointerstitial injury in animal models of DKD [35,36]. On the other hand, overexpression of several cytoprotective HSPs has been found in animal models of diabetes [37]. In our study, expression of HSP70 isoform 1B and HSP105 in HK-2 cells was increased along the time (being maximum at 48 h) by diabetic-like milieu. HSP105 has been shown as an important element of the HSP70 machinery for refolding of denatured proteins and protection against stress-induced cell death in mammalian cells [38], which suggests that overexpression of HSP105 and HSP70 in HK-2 cells might be a coordinated response against the cellular stress imposed by hypoxia and hyperglycemia.

In our study, the results related with the pathogenesis of DKD can be classified in those involving the mitochondrial reversible proton pump (F1F0-ATP synthase) and those involving changes in the expression of fibrosis-related proteins. Regarding F1F0-ATP synthase, it is well known that diabetes impairs kidney mitochondrial bioenergetics [39] and that decreased levels of ATP5B have been shown to aggravate DKD [40]. Among the different enzymes involved in the oxidative phosphorylation (OxPhos) mechanism, F1F0-ATP synthase is essential to synthesize ATP in the mitochondrion, and synthesized ATP is then exported to the cytosol by the adenine nucleotide translocase [41]. In our study, we have observed a significant decrease of ATP5B levels at 48 h and a decrease in the expression of ATP5A1 over the time, which suggests a shut-down of the OxPhos mechanism leading to a decrease in mitochondrial ATP. These results agree well with previous studies in PTC in which HG exposition reduced the ATP levels and other respiratory parameters [42, 43] or the expression of ATP synthase subunit d (ATP5PD) [44].

With regard to the expression of fibrosis-related proteins, we did not find any increase in the expression of extracellular matrix proteins (fibronectin was even decreased) in HK-2 cells exposed to hypoxia/high glucose conditions. This contrasts with the generally accepted view that deposition of extracellular matrix proteins leading to tubulointerstitial fibrosis is involved in the pathogenesis of DKD [45] and suggests that it is required more than 48 h to increase the expression of extracellular matrix proteins in HK-2 cells under diabetic-like conditions. This view may be supported by the fact that we did not find either any statistically significant difference between cells incubated in control conditions and cells incubated in diabetic-like microenvironment in the total protein/cell ratio (an index of cell hypertrophy) [46] which is expected to be increased because PTC hypertrophy is a consequence of diabetes [47]. Contrarily to extracellular matrix proteins, other changes in proteins related to renal fibrosis, i.e. cytoskeletal-related proteins [48], have been adequately detected in our experimental setting and we speculate that the decrease in the expression of TUBB, CNN2, TPM1 and ACTB in HK-2 cells exposed to the diabetic-like microenvironment might also occur in proximal tubules of diabetic patients, thereby initiating cytoskeletal alterations leading to tubulointerstitial fibrosis. The same hypothesis applies to the decreased expression of STMN1, TMSB4X and MYH9 in HK-2 cells under diabetic-like conditions, because: i) STMN1 is a microtubule-destabilizing phosphoprotein [49] whose deficiency in mice has been connected to the development of renal fibrosis [50]. In addition, STMN1 is a cell cycle regulator and its down-regulation by hypoxia has been linked to renal fibrosis in HK-2 cells through G2/M cell cycle arrest [51]; ii) TMSB4X is a potent regulator of actin polymerization with critical roles in maintaining the cell cytoskeleton [52], and daily TMSB4X treatment for 3 months reduced albuminuria and attenuated renal tubulointerstitial fibrosis in a mouse model of type 2 diabetes mellitus [53]. TMSB4X also alleviates renal tubulointerstitial fibrosis and tubular cell apoptosis in a rat model of tubulointerstitial fibrosis [54], and in tubular epithelial cells from a rat model of renal fibrosis through the TGF-β pathway [55]; iii) MYH9 is an actin regulator via activation of RhoA whose deletion in mice was associated with fibrosis and proteinuria [56]. Another cytoskeleton-related protein that might contribute to tubulointerstitial fibrosis in DKD is ezrin, which links membrane proteins to the actin cytoskeleton, and whose expression increased in HK-2 cells exposed to the diabetic-like milieu. This protein belongs to the ezrin/radixin/moesin protein family that can interact with membrane and F-actin proteins [57]. Ezrin has been suggested to contribute in PTC to the uptake by micropinocytosis of advanced glycation end products (which results from the non-enzymatic glycation of proteins during chronic hyperglycemia and contribute to DKD) [58] and thereby to promote diabetes-induced tubulointerstitial fibrosis [59]. However, further studies are needed to connect all the mentioned cytoskeletal-related changes with fibrosis in a mechanistic fashion.

It should be noted that our work has a number of limitations. We have found that exposure of HK-2 cells to the diabetic-like microenvironment results in a time-dependent increase in cell death, reaching values higher than 20% at 48 h (Fig 1A). Although the presence of some apoptotic tubular cells in both human and experimental DKD is well stablished [60–63], excessive acute cell death is not a feature of DKD. We do not know the reason of the high cell death values found *in vitro*, but even higher cell death values have been found in a previous work in which HK-2 cells or NRK-52E renal tubular epithelial cells were exposed to 30 mM glucose/1% O_2_ and apoptosis was quantified [64]. Furthermore, high glucose or hypoxia separately also induce in cultured proximal tubular cells around 15%-25% apoptotic cell death [65–70]. Thus, in DKD studies the *in vitro* models involving cultured PTC do not adequately reproduce the loss of cell viability found *in vivo*. In this connection, it should be also noted the limitations of cultured HK-2 cells in particular and of 2-dimension (2D) proximal tubular cell culture in general as models of the proximal tubule. Thus, many of the available model cell lines fail to replicate the differential expression of several uptake and efflux membrane transporters and metabolizing enzymes, which is one of the characteristics of native PTC [71]. Another major shortcoming of PTC culture is the use of metabolic fuels as compared to native proximal tubules: these reabsorb the filtered glucose and are gluconeogenic, but to drive their energy needs, native PTC rely physiologically on oxidative metabolism rather than glycolysis [72–74]. However, when PTC are brought into tissue culture, they revert from oxidative metabolism and gluconeogenesis to high rates of glycolysis. In the case of HK-2 cells, our current results and previous reports [42, 75–77], indicate that they have glycolytic and gluconeogenic activities. Interestingly, in DKD [30] and other pathological conditions, proximal tubule glycolytic flux plays an important pathogenic role [73, 78, 79, 80], which suggests that our results on glycolytic enzymes in HK-2 cells exposed to the diabetic-like milieu might be pathologically relevant. However, specific studies in native proximal tubules should confirm this hypothesis. On the other hand, it is important to note that our model has been specifically conceived to monitor the effects over time of HG and hypoxia on the protein expression in HK-2 cells and, therefore, we refer to these conditions (25 mM glucose/1% O_2_) as “diabetic-like” microenvironment rather than diabetic microenvironment because the latter is much more complex [5]. Besides, the very large and rapid time changes in glucose and O_2_ utilized here are far from being physiologic (or even pathologic, excepting in sudden vascular occlusion) and 25 mM glucose is higher than the plasma glucose concentration in most diabetic patients, whereas it is not known whether 1% O_2_ is the actual degree of hypoxia to which PTC are exposed in the kidneys of the diabetic patients. Furthermore, due to poor penetration of O_2_ through the medium, the pericellular concentration of O_2_ in cells grown in culture under atmospheric air may reach the hypoxic range [81]. On the other hand, short term exposure to HG/hypoxia may not reveal all the long-term proteomic alterations in PTC during diabetes, as suggested by our results on the expression of extracellular matrix proteins. Besides these limitations, the novelty of the present study resides on the use of a relatively simple *in vitro* model that when simultaneously exposed to hyperglycemia and hypoxia, but not exposed separately to either condition, show functional alterations that reproduce important changes induced by diabetes at the renal level in: i) the expression of glucose metabolizing enzymes ii) STAT1 and HSPs and iii) cytoskeletal-related proteins. Because of that, we believe that this model could be used as a previous step before the use of expensive *in vivo* assays.

In summary, the model we have used allows for a detailed study of the proteomic alterations induced by HG/hypoxia (i.e. two major conditions of the microenvironment that surrounds PTC in diabetes) in HK-2 cells, which may help to identify new mechanisms and targets to be investigated in the future for a better understanding of DKD. But the model has several limitations when compared with the *in vivo* situation in which native PTC are exposed to the real diabetic environment and therefore further studies in renal samples from diabetic patients or from animal models of diabetes are also required to have a better representation of the proteomic changes induced in PTC by the diabetic milieu.

## Conclusion

This is the first time-series proteomic study based on DML and UHPLC-MS/MS technology carried out to analyse the early effects of the diabetic milieu on proximal tubular cells using a model very simplified of the diabetic microambient (i.e. HG and hypoxia) that surrounds proximal tubules in diabetes. The combined exposure to HG and hypoxia conditions increased the percentage of HK-2 dead cells after 24 h without affecting the protein/cell ratio, and it significantly altered the expression of different proteins in a time-dependent manner. Several of these proteins are involved in the glycolysis pathway and their expression control seems to be HIF-1α-independent. Moreover, the abundancy of other proteins involved in the refolding of denatured proteins (HSP105 and HSP70) was also increased with time, which might be a coordinated response against the cellular stress imposed by the conditions used. On the other hand, the expression of different enzymes involved in the oxidative phosphorylation mechanism and the generation of ATP (SLC25A5, ATP5B, ATP5A1) was decreased with time, reflecting a mitochondrial dysfunction that could contribute to the development of DKD. Finally, the time expression profile showed a decrease in the expression of ten cytoskeletal-related proteins (CNN2, TPM1, STMN1 and MYH9 being reported for the first time in connection with DKD), which might be associated to renal fibrosis, but further studies are needed to connect these changes with fibrosis. With the limitations inherent to the very simplified model of the diabetic microenvironment that surrounds proximal tubules in diabetes, the present proteomic study suggests new mechanisms and targets to be investigated in proximal tubular cells in the context of the pathogenesis of DKD.

## Supporting information

S1 Table Raw data for protein identification and quantification. (XLSX)

S2 Table Proteins included in the different clusters obtained using Mfuzz toolbox, and the pathways/molecular functions in the PANTHER website (http://www.pantherdb.org/) in which they are involved. (XLSX)

S3 Table Proteins included in the different clusters obtained using Mfuzz toolbox, and the pathways/molecular functions in the STRING website [26] in which they are involved. (XLSX)
